# A Rare Case of Missing Primary in Metastatic Renal Cell Carcinoma

**DOI:** 10.7759/cureus.8637

**Published:** 2020-06-15

**Authors:** Ankita Kapoor, Nikhil Khushalani, Shipra Gandhi

**Affiliations:** 1 Internal Medicine, Rochester General Hospital, Rochester, USA; 2 Cutaneous Oncology, H. Lee Moffitt Cancer Center and Research Institute, Tampa, USA; 3 Oncology, Roswell Park Cancer Institute, Buffalo, USA

**Keywords:** renal cell carcinoma, sunitinib, radiographic primary, metastatic rcc, papillary rcc

## Abstract

Renal cell carcinoma (RCC) can present with a myriad of clinical symptoms and signs. It is also notorious for its initial presentation with distant metastasis. We report a case of a 42-year-old male diagnosed with papillary RCC (PRCC) presenting with pleural and nodal metastases in the absence of a radiographically-detected tumor primary. PRCC was diagnosed on immunohistochemical analysis of the tissue from the pleura and mediastinal lymph nodes and confirmed by gene expression profiling studies. As per treatment guidelines for metastatic RCC, the patient was started on sunitinib with evidence of disease progression after two cycles and palliative care approach was recommended due to rapidly declining performance status. Prospective data on the optimal management of metastatic PRCC are lacking, but drugs used are similar to the treatment of clear cell carcinomas (vascular endothelial growth factor (VEGF) tyrosine kinase inhibitors, mammalian target of rapamycin inhibitors) and checkpoint inhibitors. Further molecular study of these rare tumors is warranted to detect drivers of oncogenesis and identify targets for therapeutic intervention.

## Introduction

Renal cell carcinoma (RCC) is amongst the most common genitourinary malignancies and is notorious for its initial presentation with distant metastases [1]. Approximately 85% of kidney tumors are RCC, and approximately 70% of these have a clear cell histology. Other less common types include papillary, chromophobe, translocation, and Bellini duct (collecting duct) tumors [1-3]. Metastatic lesions from RCC have been found in almost every organ or tissue of the body. Histopathologic evaluation of the metastatic lesion in a patient with asymptomatic RCC with signs and symptoms due to distant metastases, reveals the diagnosis.

There are very few case reports in the literature about RCC metastasis without documented evidence of a radiographical primary. Because of a better understanding of the molecular pathways implicated in RCC pathogenesis, there has been a transition in the treatment of RCC from nonspecific cytokine-based immune approach, to targeted therapy against vascular endothelial growth factor (VEGF) and mechanistic target of rapamycin (mTOR) to checkpoint inhibitors [4]. We present a case of a 42-year-old male diagnosed with metastatic papillary RCC (PRCC) to the pleura and the lymph nodes without radiographic evidence of a renal primary.

## Case presentation

**Figure 1:**
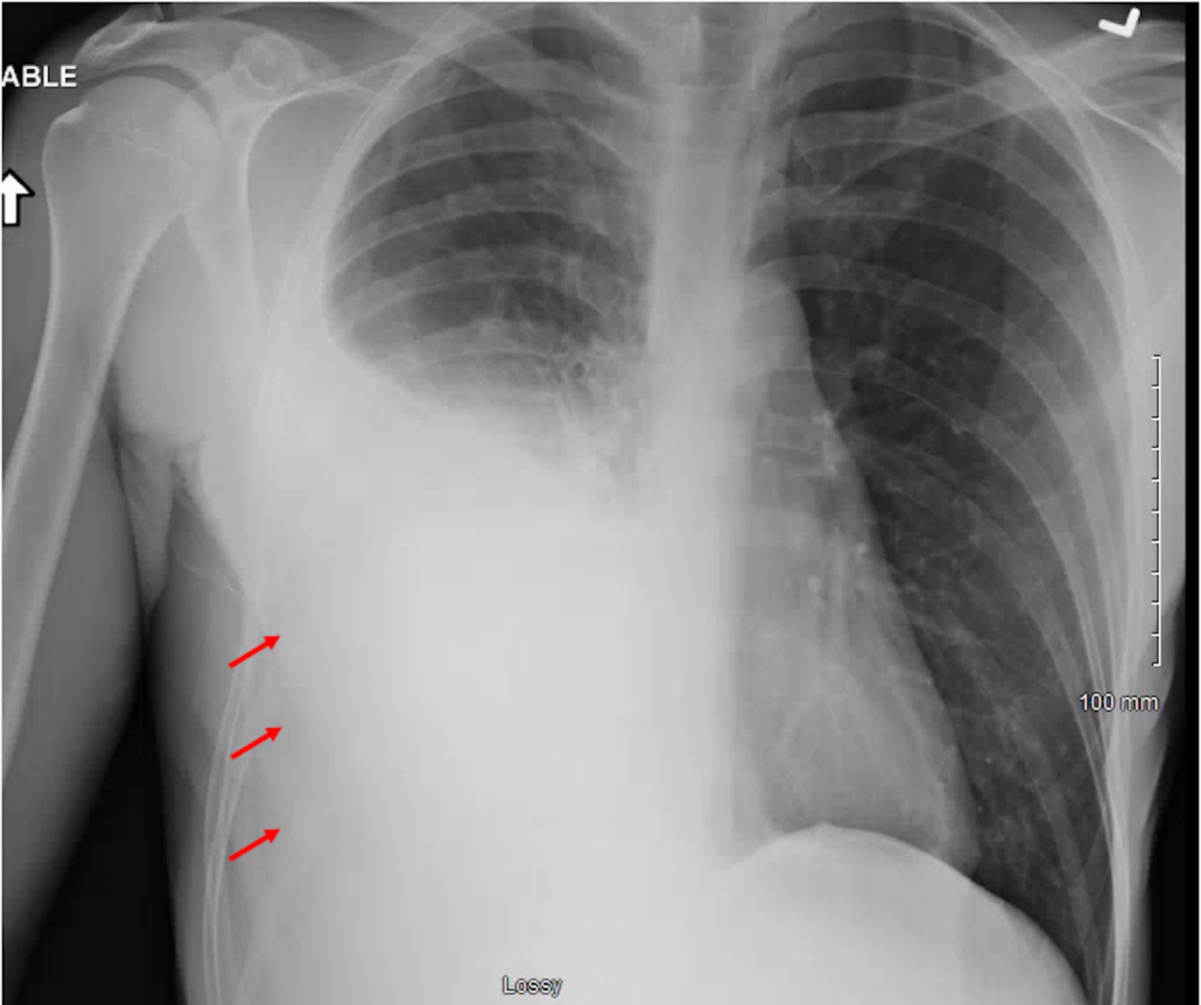
Chest X-ray revealing right-sided pleural effusion

**Figure 2:**
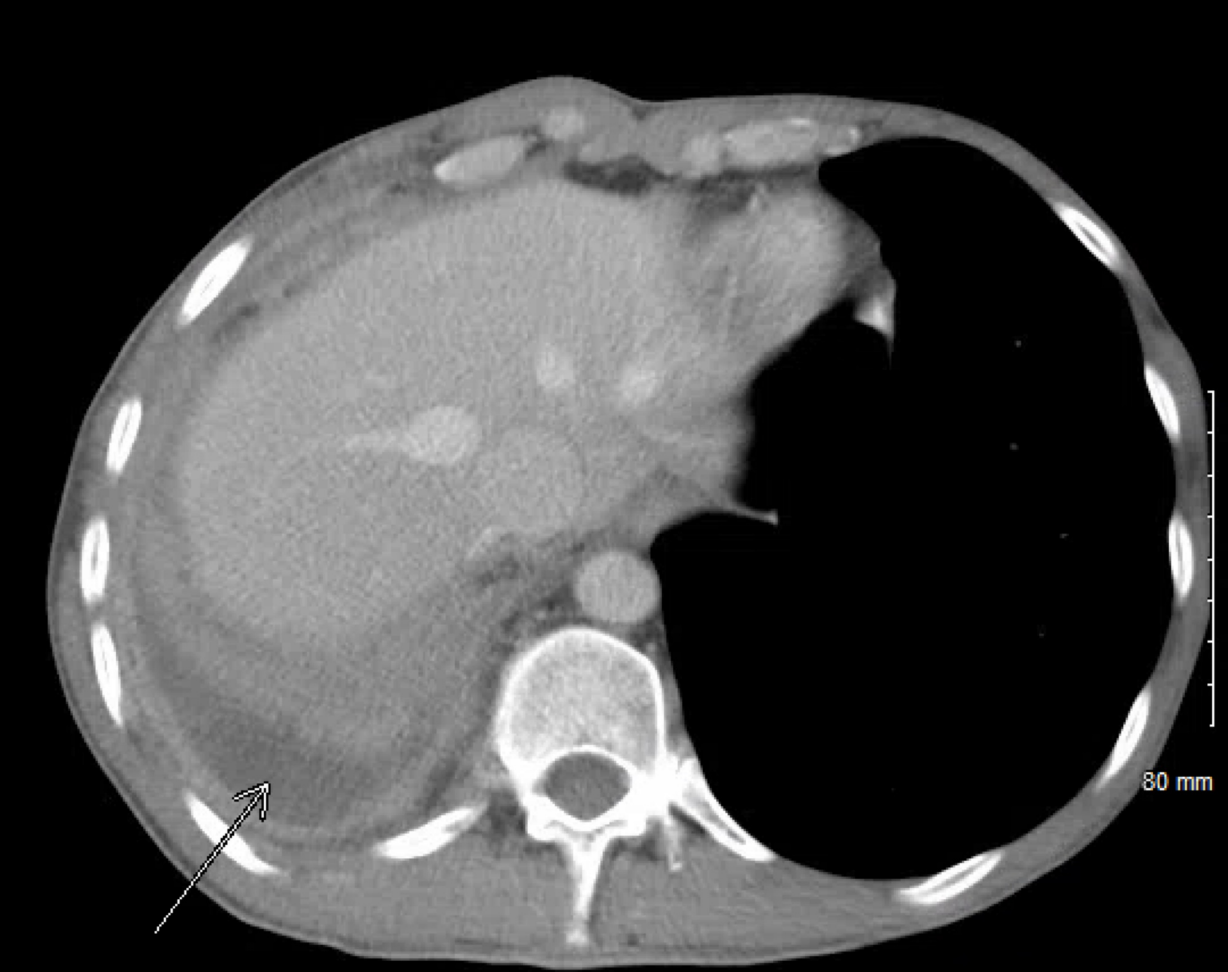
CT chest showing loculated pleural effusion on right side indicated by arrow

**Figure 3:**
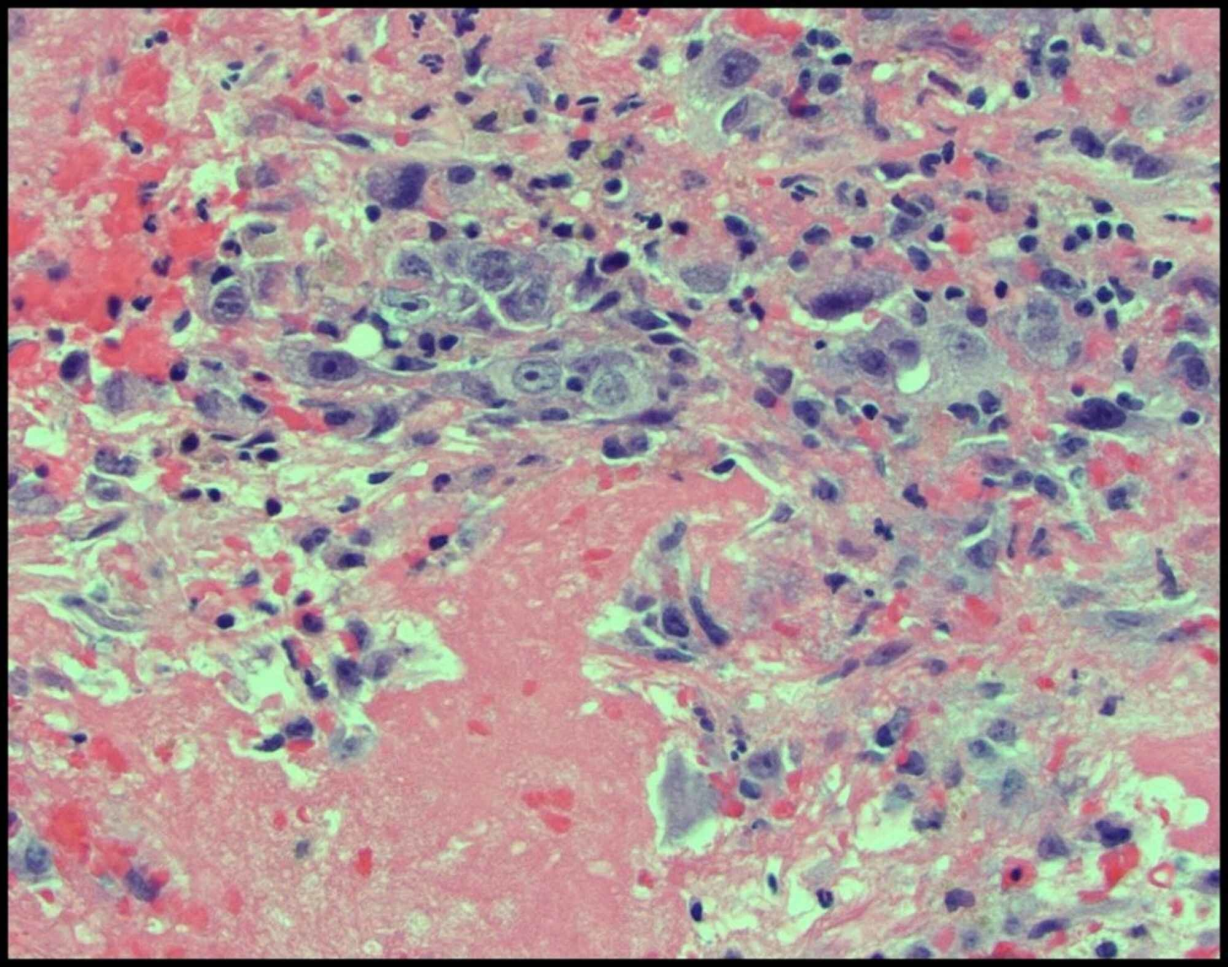
Pleural fluid showing highly atypical epitheloid cells with granular cytoplasm, vesicular nuclear chromatin and prominent eosinophilic nucleoli

**Figure 4:**
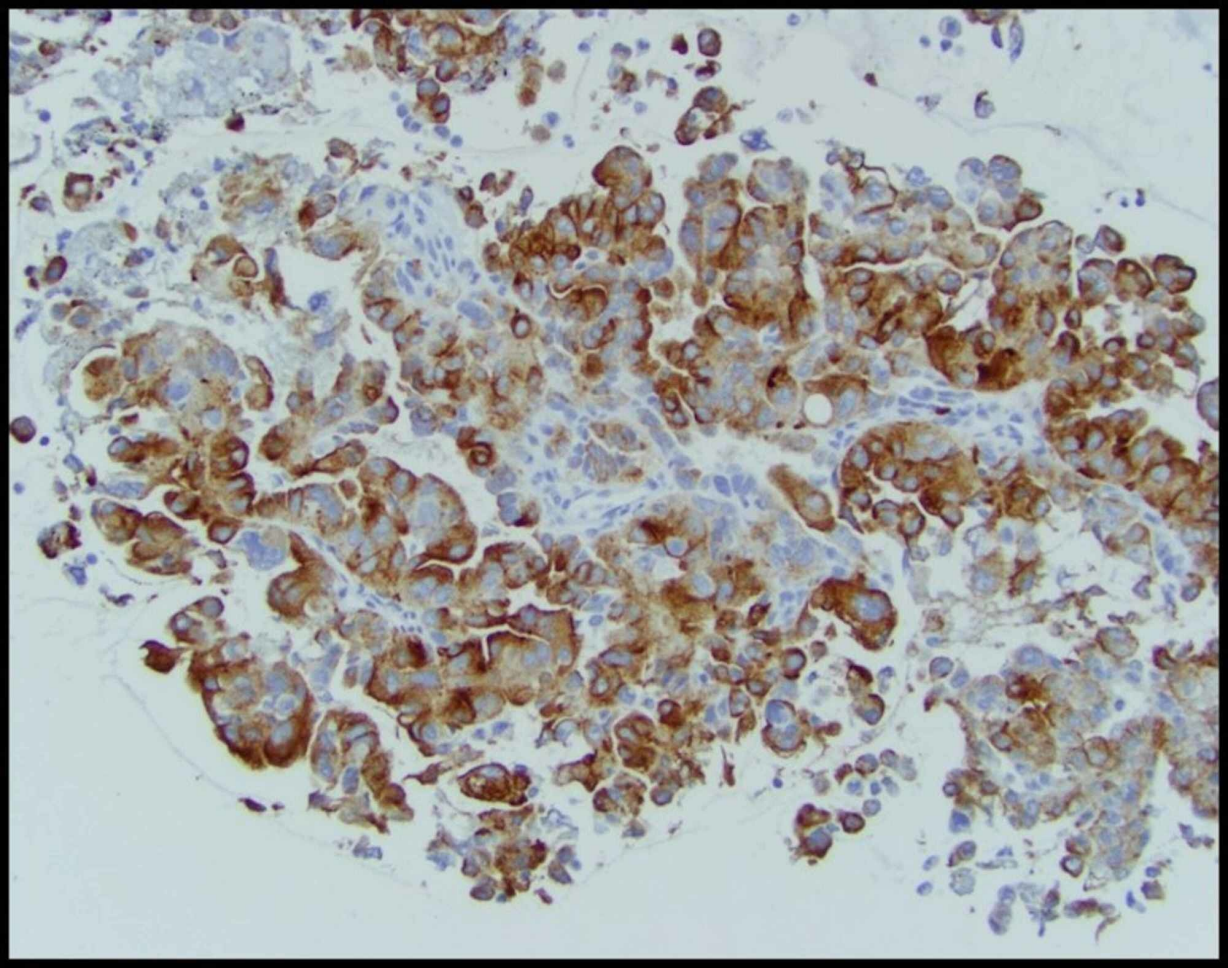
Renal cell carcinoma (RCC) antigen expression in paratracheal lymph node

**Figure 5:**
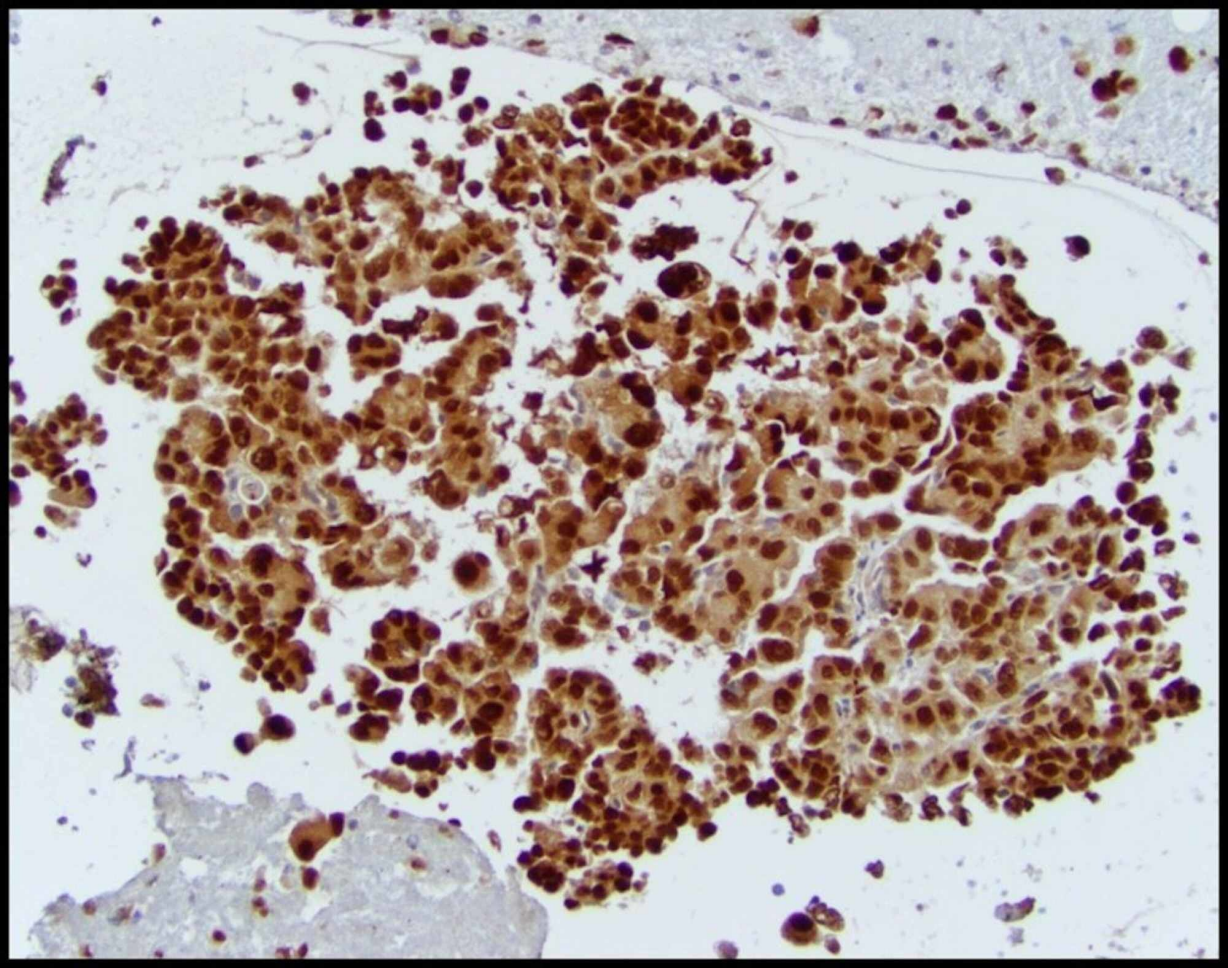
Paired box-8 (PAX-8) expression in paratracheal lymph node showing strong nuclear and weak cytoplasmic immunoreactivity

**Figure 6:**
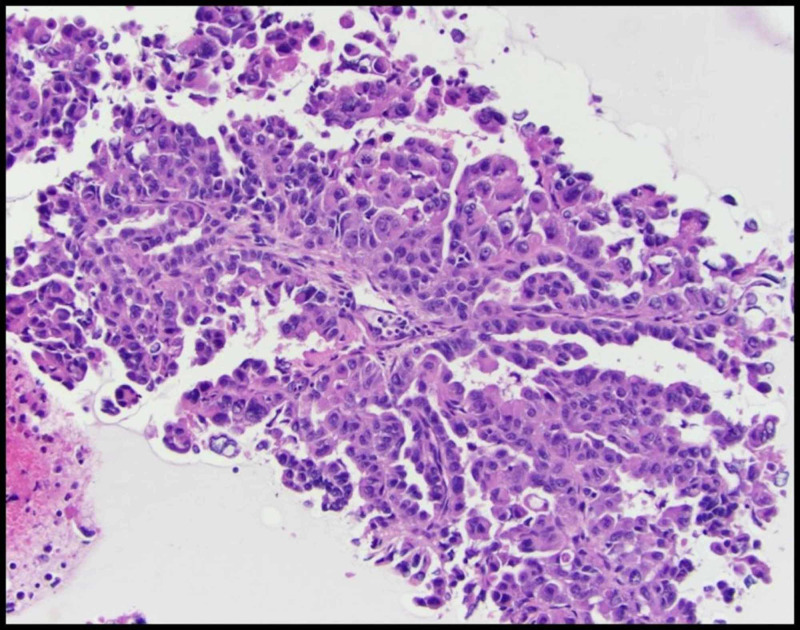
Paratracheal lymph node showing a papillary tumor

**Figure 7:**
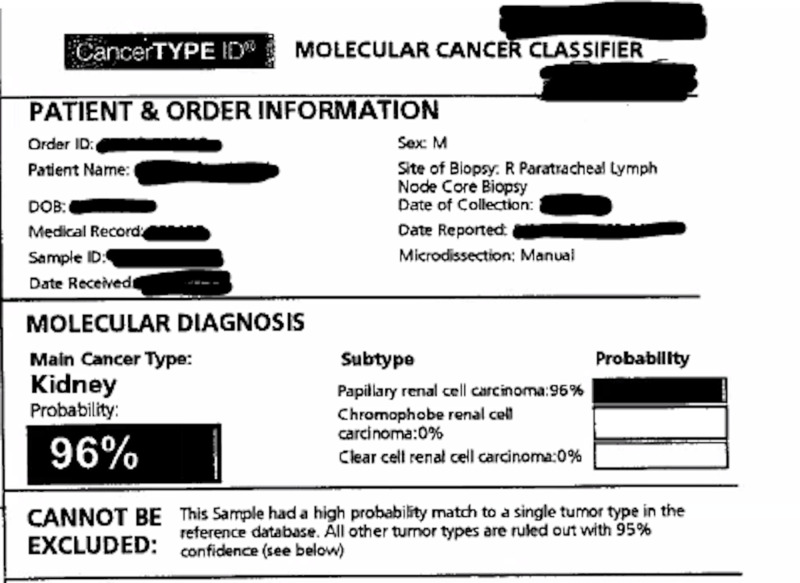
Reverse transcription-polymerase chain reaction (RT-PCR) confirming 96% probability of papillary renal cell carcinoma

**Figure 8:**
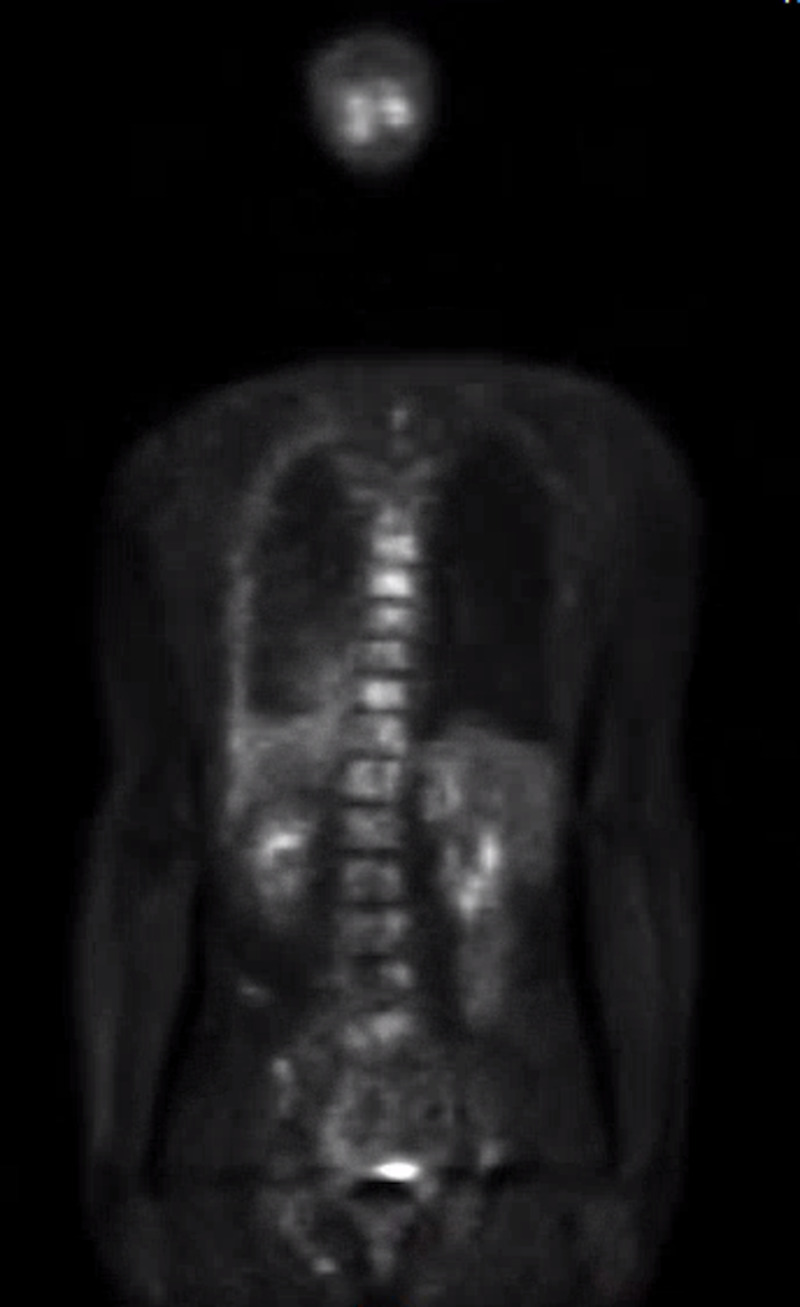
Positron emission tomography/computed tomography (PET/CT) did not reveal primary renal cell carcinoma

**Figure 9:**
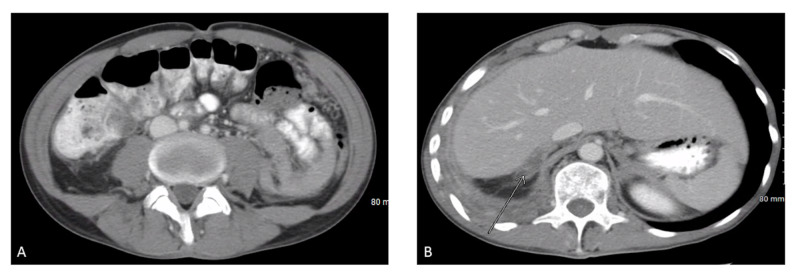
CT abdomen after two cycles of sunitinib treatment showing disease progression Figure A reveals peritoneal carcinomatosis and Figure B reveals pleural metastatic disease marked by an arrow.

We report a case of a 42-year-old male with 10 pack-year smoking history who presented with right-sided chest pain following a fall. Chest X-ray revealed right pleural effusion which failed to resolve upon treatment for pneumonia with antibiotics (Figure 1). Subsequent CT-scan showed right-sided loculated effusion with the collapse of the right middle and lower lobes with mediastinal adenopathy (Figure 2). This prompted thoracentesis which drained 1.2 liters of hemorrhagic fluid, likely right-sided hemothorax, possibly, secondary to trauma. Since the hemothorax did not resolve upon chest tube placement, video-assisted thoracoscopic surgery (VATS) with total decortication was performed and pathological analysis revealed highly atypical epithelioid cells with granular cytoplasm, vesicular nuclear chromatin and prominent eosinophilic nucleoli, differentials being pleural epithelial angiosarcoma v/s reactive pleural cells v/s metastatic PRCC (Figure 3). For further diagnostic clarification, endobronchial ultrasound (EBUS)-guided mediastinal lymph node biopsy demonstrated metastatic carcinoma with immune profile positive for epithelial membrane antigen (EMA), cluster of differentiation 10 (CD10), RCC, paired box 2 (PAX-2), PAX-8, CK A/E 1/3, vimentin, Ber EP-4, Moc-31, and negative for mucicarmine, thyroid transcription factor-1 (TTF-1), cytokeratin 7 (CK 7), CK 5/6, p63 and thus being most consistent with metastatic RCC (Figures 4-5) [5]. The cytomorphology features were consistent with papillary carcinoma (Figure 6). A 92-gene reverse transcription-polymerase chain reaction (RT-PCR) assay on the tissue also confirmed a 96% probability of PRCC (Figure 7). Positron emission tomography/computed tomography (PET/CT) showed no evidence of thyroid, colorectal, and renal neoplasm with fluorodeoxyglucose (FDG) avidity noted in the mediastinum and right pleura (Figure 8). The diagnosis of metastatic RCC was confirmed by two pathologists on two different specimens, that is, lymph node and pleura. Considering the histology and immune profile, the patient was diagnosed with metastatic PRCC to the pleura and the mediastinal lymph nodes in the absence of a discernable primary renal lesion. A consensus decision was reached to treat the patient along the lines of a RCC considering the pathology, despite the lack of radiographic detection of a renal primary. The patient was started on sunitinib, a tyrosine kinase inhibitor, the standard treatment for metastatic RCC. However, after two cycles, a restaging CT showed progressive disease with extensive omental/peritoneal carcinomatosis, as well as the development of widespread bony, adrenal, and brain metastases (Figure 9). Because of the patient’s rapidly progressive disease course with decline in performance status and failure to thrive, palliative approach was taken and the patient passed away. 

## Discussion

RCC presents with myriad of clinical manifestations. Only 10% of patients present with the classic triad of hematuria, flank pain, and palpable mass. Around 30% of all cases are asymptomatic and are detected during the course of various diagnostic investigations.Around 25% of patients with RCC have distant metastatic lesions at the time of initial presentation [1].A wide array of metastatic sites have been described. More commonly, RCC metastasizes to lungs, followed by liver, bone, brain, soft tissue, pleural spaces. They are also notorious to metastasize to bones, joints, breast and genitourinary organs. These tumors being highly vascular have high metastatic potential as they express VEGF, vascular endothelial growth factor receptor (VEGFR), basic fibroblast growth factor (bFGF), and platelet derived growth factor (PDGF) [6]. Additionally, the main mechanism of systemic metastases is through hematogenous spread as the kidney receives 25% of circulating blood volume per minute [7]. Our patient presented with metastasis to the pleura and mediastinal lymph nodes without a primary source which led us to think of the possible primaries that frequently metastasize to the pleura. A study conducted by Meyer et al. in 1966 on metastatic carcinomas to the pleural cavity revealed that more than half of the carcinomas metastatic to the pleura were of bronchial origin [8]. There are numerous mechanisms by which a primary malignancy can metastasize to the pleura - metastasis via pulmonary arterial tumor emboli (bronchial cancer), tertiary spread via hepatic secondaries, direct chest wall invasion (breast cancer). Effusions can also develop as a result of neoplastic infiltration of the mediastinal lymph nodes and are not related to the extent of pleural involvement by the nodular metastasis. Pleural metastases is comparatively rarer than lung metastases in RCC [8]. There are case reports in the past with metastatic RCC to supraclavicular lymph nodes, digits, head, and neck, skin in the absence of a discernable primary [9]. In a review, Koga et al. reported that 18 of the 75 cases of cutaneous RCC in Japanese reports had lesions with no known primary [10]. However, in some of these cases, years later, a renal primary appeared. It is not clear why the primary lesion remains occult, but it may be involuted or too small to be detected. Close monitoring is essential as these lesions could be silent renal tumors [9].

A panel of immunohistochemical markers are used for diagnosis of metastatic RCC, which often include CK-7, CK-20, CD10, vimentin, and PAX2 [5,11]. CK7 and CK20 panel is frequently applied as initial screening panel for metastatic investigation to reflect the most likely tumor gene site. In 80% of cases, it is positive for both CK7 and CK20 although, in 17%, it is CK7+/CK20- and while in 3% it is, CK-7-/CK-20+[5]. In our case, histological analysis of pleura revealed high grade and poorly differentiated tumor cells exhibiting highly atypical vesicular chromatin with multiple nuclear grooves and prominent macronuclei. Considering the papillary nature of the cells, the differentials included metastasis from thyroid, kidney, ovary, breast or pancreas. However, in the absence of radiographic evidence of a primary lesion; immunostaining served as a valuable tool to determine the type of tumor [12]. On immunohistochemistry, the tumor cells were positive for RCC, CD10, PAX-2, PAX-8, vimentin and negative for mucicarmine, TTF-1, CK-7, and p63. Thus the immunoprofiling was consistent with metastasis from a renal PRCC as a primary [5,11].

Non-clear cell RCC accounts for 25%-30% of all RCCs and has distinct molecular characteristics, histologies, and clinical outcomes. This group includes papillary RCC which was seen in our patient. In addition, RT-PCR also confirmed the diagnosis of PRCC. Once metastatic, non-clear cell RCC are usually resistant to systemic therapies that are active against clear cell RCC but still the general approach is to use the same options which are used for clear cell RCC [13,14]. Over the past decade, there have been considerable advances in the treatment of advanced RCC because of better understanding of molecular pathways implicated in pathogenesis. In the pre-targeted therapy area, cytokine based immunotherapy either with interleukin-2 (IL-2) or interferon-alfa (IFN-α) or a combination of both who were regarded as standard of care for advanced RCC even with known significant toxicities and low response rates [15]. In the last decade, there has been a rapid development of novel molecular targeted agents due to identification of VEGF, VEGFR and mTOR as dysregulated signaling pathways in the development and progression of RCC. Targeted therapies, including sorafenib, sunitinib, bevacizumab (in combination with IFN-γ), temsirolimus, everolimus, have been evaluated in randomized, controlled phase III clinical trials of patients with metastatic RCC and have been approved by the US Food and Drug Administration (FDA) for the management of metastatic RCC [16]. The best data supporting sunitinib has been based on two randomized, phase two clinical trials, ASPEN and ESPN which included total of 176 patients and amongst them 55% had papillary RCCs. Overall progression-free survival (PFS) was longer for sunitinib in both studies (8.3 vs 5.6 months, and 6.1 vs 4.1 months) and overall survival (OS) was longer for sunitinib in ESPN study (16.2 vs 14.9 months) compared to everolimus [17]. Currently, the default standard of care for non-clear cell RCC is VEGF targeting agents, although new clinical trials are strongly encouraged because of poor outcomes. Novel immunotherapy with checkpoint inhibitor nivolumab has become an established modality in clear cell RCC. However, clinical trials with nivolumab have excluded patients without a clear‐cell component. In a study of 101 patients with non-clear cell RCC, PD‐L1 was expressed in 10.9% and was more common in the papillary (30%) and translocation (20%) subtypes. Patients who had tumors with PD‐L1 expression appeared to have worse outcomes (P = .08), but that may have been because of higher tumor stage and grade [18]. Recent developments have raised the question of whether patients benefit most from combinatorial or sequential therapy of targeted agents. At present, sequential therapy with targeted agents with different mechanisms and minimal cross-resistance is the standard of care. European Association of Urology Guidelines Group for RCC has published the latest evidence-based treatment guidelines and considers VEGFR-TKI sunitinib as the standard of care for first-line therapy for patients with low- or intermediate-risk metastatic RCC [19].

As in our case, the patient was initially started on sunitinib, however, due to disease progression, a consensus decision was reached to switch the treatment to temsirolimus and observe the tumor response. However, temsirolimus could not be initiated because of the rapid decline in functional status.

## Conclusions

To our knowledge, this is the first case of metastatic PRCC to the pleura in the absence of a radiographically detected primary tumor. The appearance of a primary renal tumor in the course of follow-up has been documented on previous rare reports. This case highlights the dilemmas in diagnostic pathology and stresses the importance of modern immunohistochemical and molecular techniques aiding the accurate diagnosis of carcinoma of an unknown primary origin. This may be useful in prognostication and in streamlining management paradigms for these patients. Prospective data on the optimal management of metastatic PRCC are lacking, but drugs used are similar to the treatment of clear cell carcinomas (VEGF tyrosine kinase inhibitors, mammalian target of rapamycin inhibitors) and checkpoint inhibitors. Further molecular study of these rare tumors is warranted to detect drivers of oncogenesis and identify targets for therapeutic intervention. 
